# Perivascular cells and tissue engineering: Current applications and untapped potential

**DOI:** 10.1016/j.pharmthera.2016.11.002

**Published:** 2017-03

**Authors:** Elisa Avolio, Valeria V. Alvino, Mohamed T. Ghorbel, Paola Campagnolo

**Affiliations:** a Division of Experimental Cardiovascular Medicine, Bristol Heart Institute, University of Bristol, United Kingdom; b Division of Congenital Heart Surgery, Bristol Heart Institute, University of Bristol, United Kingdom; c School of Biosciences and Medicine, University of Surrey, Guildford, United Kingdom

**Keywords:** Biomaterials, Pericytes, Perivascular cells, Tissue engineering, Vascular graft, Scaffold vascularization, 3D, 3-dimensional, AFs, angiogenic factors, Ang1/2, angiopoietin 1/2, BBB, brain blood barrier, BFs, bioactive factors, BMSCs, bone marrow mesenchymal stem cells, BPs, brain pericytes, CPs, cardiac pericytes, CRBP1, cell retinol binding protein, ECM, extracellular matrix, ECs, endothelial cells, EPCs, endothelial progenitor cells, ePFTE, polytetrafluoroethylene, FGF, fibroblast like growth factor, GFAP, glial fibrillary acidic protein, GFs, growth factors, HUVECs, human umbilical vein endothelial cells, iPS, induced pluripotent stem cells, LPs, liver pericytes, MHC-I/II, major histocompatibility complex I/II, MI, myocardial infarction, MPs, myocardial pericytes, MSCs, mesenchymal stem cells, NG2, neural/glial antigen 2, PCL, polycaprolactone, PCs, perivascular cells, PDGF, platelet derived growth factor, PDGFR-β, platelet derived growth factor receptor beta, PEG, poly(ethylene glycol), PGA, polyglicolyc acid, PLGA, poly(dl-lactide-co-glycolic acid), PLLA, poly-l-lactic acid, RGS5, regulator of G-protein signaling 5, SkPs, skeletal muscle pericytes, SM-MHC, smooth muscle myosin heavy chain, SMCs, smooth muscle cells, SMemb, non-muscle myosin heavy chain IIB, SVPs, saphenous vein derived pericytes, TE, tissue engineering, TEVG, tissue engineered vascular graft, TGFβ, transforming growth factor beta, UCPCs, umbilical cord derived perivascular cells, VEGF, vascular endothelial growth factor, vWF, von Willebrand Factor, α-SMA, smooth muscle alpha-actin

## Abstract

The recent development of tissue engineering provides exciting new perspectives for the replacement of failing organs and the repair of damaged tissues. Perivascular cells, including vascular smooth muscle cells, pericytes and other tissue specific populations residing around blood vessels, have been isolated from many organs and are known to participate to the *in situ* repair process and angiogenesis. Their potential has been harnessed for cell therapy of numerous pathologies; however, in this Review we will discuss the potential of perivascular cells in the development of tissue engineering solutions for healthcare. We will examine their application in the engineering of vascular grafts, cardiac patches and bone substitutes as well as other tissue engineering applications and we will focus on their extensive use in the vascularization of engineered constructs. Additionally, we will discuss the emerging potential of human pericytes for the development of efficient, vascularized and non-immunogenic engineered constructs.

## 1 Introduction

### 1.1 Perivascular cells: types, characterization and function

Perivascular cells (PCs) can be isolated from multiple tissues in the body and play a role in tissue repair, vascular homeostasis as well as angiogenesis. Perivascular cells include mainly two types of cells: vascular smooth muscle cells (SMCs) and pericytes. While SMCs surround large vessels such as arteries and veins and are separated from endothelial cells (ECs) by the basement membrane and inner elastic lamina, pericytes surround smaller vessels and capillaries and are in direct contact with ECs (Crisan, Corselli, Chen, & Peault, 2012).

SMCs are a mixed cell population with a complex developmental origin (Owens, Kumar, & Wamhoff, 2004), their marker expression profile has been thoroughly studied. During their synthetic and proliferative phase SMCs typically express vimentin, non-smooth muscle myosin heavy chain IIB (SMemb), tropomyosin 4 and cell retinol binding protein (CRBP1); during maturation, quiescent SMCs express increasing levels of smooth muscle alpha actin (α-SMA), transgelin and basic calponin, and finally smooth muscle myosin heavy chain (SM-MHC) at late stages of development (Wanjare, Kusuma, & Gerecht, 2013).

Pericytes have been more recently isolated, and while many common markers have been identified, a consensus has not yet been reached. In particular, pericytes display different expression patterns depending on their tissue of origin, the activation state and culture/expansion protocol, and whether the characterization is carried out *in vivo* or *in vitro*. Furthermore, different nomenclatures have been developed to describe the various subtypes of micro-perivascular cells. Pericytes possess similar properties with mesenchymal stem cells (MSCs), such as the antigenic expression of CD44, CD90, CD105, the lack of hematopoietic (CD45) and endothelial (CD31, von Willebrand Factor – vWF, VE-Cadherin) markers, multipotential differentiation capacity (adipogenic, osteogenic, chondrogenic and myogenic differentiation), clonogenicity, immunosuppression, non-alloreactivity, wound healing contribution and extracellular matrix (ECM) regeneration. Indeed, it has been hypothesized that pericytes could represent a tissue-resident perivascular source of MSCs (Campagnolo et al., 2010, Corselli et al., 2013, Hass et al., 2011, Kovacic and Boehm, 2009).

**Fig. 1:**
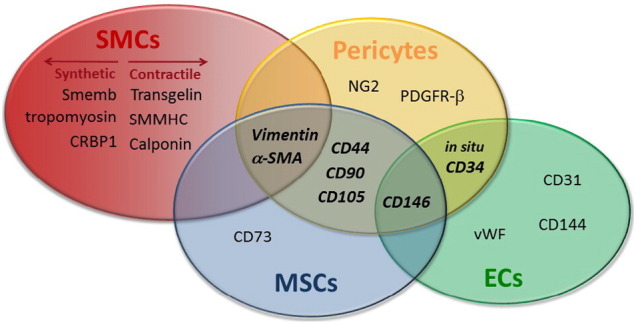
Venn diagram showing the overlap of markers expression between different classes of vascular and perivascular cells. Markers shared by 2 or more cell types are indicated with bold and italic characters.

A summary of the most commonly described markers and their co-expression by related cell types is reported in Table 1. It is important to highlight the partial overlapping in the expression of markers between different cell types, as clarified in Fig. 1 (Covas et al., 2008, Valente et al., 2014). The classification of the mesodermal derived lineages is the topic of heated ongoing debate; while beyond the scope of this review to provide extensive definition of the cell identity, we look forward to the definition of a more precise immunophenotype characterization for these cell types.

This review will focus on the tissue engineering (TE) applications of perivascular cells: SMCs, pericytes and pericyte-associated cells. We will provide a summary of the main studies involving the use of perivascular cells for vascular, skeletal and cardiac muscles, bone and dermal TE. Since the description and definition of SMCs is well established, we will give an introductory overview of the several populations of pericytes and pericyte-associated cells that have been used for TE purposes.

### 1.2 Definition and characterization of pericytes from different sources

Pericytes are perivascular cells that surround ECs in capillaries, venules and arterioles; their shape, size, distribution, attachment and density depend on their location. Pericytes derived from different body districts share similar phenotype and functional properties; however, since the nomenclature of tissue-derived microvessel perivascular cells is still debated, we remand this issue to a more specialized article. For the scope of this review the term *peri-microvascular cells* or *pericytes* will be adopted to indicate several of the populations so far described: these include both (i) *de facto* pericytes, surrounding microvessels of different tissues and (ii) adventitial pericyte-associated cells found within the *vasa vasorum* of vein and arteries and in the heart tissue (Avolio et al., 2015a, Campagnolo et al., 2010, Chen et al., 2015, Corselli et al., 2013, Kovacic and Boehm, 2009).

It is general consensus that most pericytes express neural/glial antigen 2 (NG2) and platelet-derived growth factor receptor beta (PDGFRβ) and lack the expression of hematopoietic and endothelial markers, such as CD45 and CD31 (Campagnolo et al., 2010, Chen et al., 2015, Crisan et al., 2008). A summary of the expression profile of pericytes and pericyte-associated cells in relation to their source and strategy of isolation is reported in Table 2.

In terms of function, the general role of pericytes is the control of vascular permeability, however cells from different districts have shown remarkably different characteristics, which can be exploited for specific TE applications.

Brain pericytes (BPs) constitute an important part of the blood brain barrier (BBB) by sequestering small molecules before they reach the extracellular fluid of the brain (Bouchard, Shatos, & Tracy, 1997). This ability has been harnessed for engineering a BBB model where astrocytes, pericytes and ECs are placed in a 3-dimensional (3D) hydrogel matrix of collagen type I (Tourovskaia, Fauver, Kramer, Simonson, & Neumann, 2014).

Liver pericytes (LPs) participate in the vitamin A (retinol) metabolism, the repair of hepatic tissue through the recruitment of inflammatory cells and the ECM remodeling through the secretion of degrading enzymes - metalloproteinases (MMPs) - and their inhibitors (Sims, 2000). LPs are involved in diseases such as cirrhosis, hypertension of portal vein and hepatic cancer, as well as in their treatment. In addition, LPs have been used in TE applications such as the repopulation of decellularized human liver matrix, showing excellent viability, motility, proliferation and remodeling of ECM (Mazza et al., 2015).

Saphenous vein-derived CD34 +/CD146- adventitial pericytes showed remarkable pro-angiogenic capacity when injected directly into an ischemic area, both in hindlimb ischemia and in myocardial infarction. These cells were able to migrate into damaged site, stimulate the angiogenesis through direct contact with ECs, and contribute to the neo-angiogenesis and blood flow restoration (Avolio et al., 2015b, Campagnolo et al., 2010, Gubernator et al., 2015, Katare et al., 2011).

CD146 + pericytes were isolated from skeletal muscle (SkPs) and several other human tissues, including pancreas, adipose tissue and placenta. As they present a remarkable myogenic ability, Crisan et al. have exploited their characteristics for muscle regeneration. Briefly, these cells, purified using fluorescent activated cell sorting, can been cultured in a muscle proliferation medium to form myotubes and contribute to muscle regeneration when injected in a mouse model of muscular dystrophy (Chen et al., 2015, Crisan et al., 2008, Park et al., 2011).

Adipose tissue is a useful source of cells for regenerative medicine purposes due to its abundance and easiness of harvesting. Several multipotent populations associated with the micro-vascular niche have been isolated and described. Both CD34 positive and negative populations were described as residing perivascularly and exhibiting pericyte-like markers (NG2, PDGFRb), with the CD34- fraction expressing the *in sensu stricto* pericyte marker CD146 (Crisan et al., 2012, Traktuev et al., 2008, Zannettino et al., 2008). Interestingly, some of these populations display characteristics useful in the context of regenerative medicine, such as promoting the recovery of hind-limb ischemia (Miranville et al., 2004) and bone reconstruction (Zannettino et al., 2008) in murine models. Umbilical cord perivascular cells (UCPCs) represent an interesting population for TE due to their easy accessibility and availability. UCPCs are CD146 +, clonogenic, highly proliferative, immunosuppressive and capable of differentiation into the mesenchymal lineages. Additionally, UCPCs were able to efficiently engraft in the defective bone, indicating their suitability for bone regeneration (Sarugaser et al., 2005, Tsang et al., 2013).

Dental pulp tissue contains a perivascular niche with odontoblast-like progenitor cells that co-express CD146 and STRO-1, an osteogenic precursor marker (Alliot-Licht et al., 2005, Shi and Gronthos, 2003).

More recently, pericyte-like cells have also been isolated from the human heart. They are clonogenic and committed toward the vascular SMCs lineage and secrete a variety of pro-angiogenic and chemotactic factors able to attract cardiac progenitor cells and ECs (Avolio, Rodriguez-Arabaolaza, et al., 2015). In the same year Chen and colleagues isolated a population of myocardial pericytes (MPs) from fetal and post-mortem adult myocardial samples. MPs are able to differentiate into cardiomyocyte-like cells both *in vitro* and *in vivo* when transplanted in infarcted mouse hearts (Chen et al., 2015).

Pericytes have been derived also from human induced pluripotent stem cells (iPS) following multi-step differentiation protocols (Dar et al., 2012, Kusuma et al., 2015, Kusuma and Gerecht, 2016, Orlova et al., 2014b, Wanjare et al., 2014). The application of stem cell derived pericytes in TE has been suggested and is probably under investigation, the main advantage being the easy availability and the potential to obtain patient-derived cells through induction of pluripotency in somatic cells (Dar & Itskovitz-Eldor, 2015). Indeed, the obtained pericytes are functionally competent, as demonstrated by the cooperation with other vascular cells during the formation of vascular-like structures *in vitro* (Kusuma et al., 2014, Orlova et al., 2014a) and their angiogenic capacity *in vivo* (Dar et al., 2012).

Overall, thanks to their capacity to stabilize blood vessels, regulate angiogenesis and immunological response and contribute to physiological and pathological repair processes, perivascular cells are great candidates for TE applications (Gokcinar-Yagci, Uckan-Cetinkaya, & Celebi-Saltik, 2015).

## 2 Tissue engineering

The development of cell therapy has improved the therapeutic options for many diseases. So far, the majority of preclinical studies and clinical trials have focused on the delivery of cells suspensions by injection in the area of damage; however, the benefits of cell therapy have been limited by the poor survival and rapid removal of cells (Naderi-Meshkin, Bahrami, Bidkhori, Mirahmadi, & Ahmadiankia, 2015). Evidence shows that the injected cells do not contribute to the reconstitution of the damaged tissue, highlighting the urgency of new solutions for organ/tissue replacement. Based on these considerations, clinicians and biologists are developing new techniques in the attempt to generate biological tissues (“grafts”) *in vitro*, developing the new field of TE.

The reconstruction of tissues can be achieved by the combination of a support material (“scaffold”) with cells and/or bioactive factors (BFs).

The scaffold can be of natural or synthetic origin and is meant to provide support to the forming tissue and a matrix for cell retention and controlled BF release. Natural matrices are made of biologically-derived polymers, such as collagen, elastin, fibrin, fibronectin, alginates electrospun or made into a hydrogel. Alternatively, they can consist of entire decellularised tissues, commonly xenografts of porcine or bovine origin. Conversely, synthetic matrices are composed of synthetic polymers like polyglicolyc acid (PGA), poly(dl-lactide-co-glycolic acid) (PLGA), poly-l-lactic acid (PLLA) and polycaprolactone (PCL) (extensively revised in Keane and Badylak, 2014, Lee et al., 2014, Li et al., 2015, Vashist and Ahmad, 2015).

On the one hand, biological matrices have the advantage of providing an adequate anatomic structure (for example the porcine decellularised valve) and are bioactive, stimulating the recruitment of cells to the graft. On the other hand, however, synthetic matrices are available in limitless supply and allow tailoring the material characteristics to meet the desired porosity (pores dimension), topography (surface characteristics, for example to promote cell adhesion) and mechanical properties according to the organ in which the graft will be implanted.

The addition of cells to the scaffold structure contributes to the mechanical stability through the release of ECM. Furthermore, the seeded cells provide an immediate non-immunogenic surface and release a plethora of growth factors (GFs) attracting host cells and regulating the inflammatory response. Also, the cellular component promotes the creation of a living tissue that is integrated with the recipient organ and accelerates the full achievement of the therapeutic activity. The cells are selected based on the desired functional properties, with autologous cells being the preferred choice for clinical applications to avoid immunosuppression of patients.

Last, BFs can be integrated within the biomaterial to improve the biocompatibility or create chemoattractant gradients and recruit cells to the graft or to support cell survival, accelerating the formation of a functional tissue (Andreas, Sittinger, & Ringe, 2014).

In addition, there is also an alternative TE strategy, named scaffold-free, based on the generation of cell sheets *in vitro* through stacking of multiple layers of cells together. This is the case, for example, of cardiac patches (Sakaguchi, Shimizu, & Okano, 2015).

## 3 Vascularization of biomaterials

To date, one major obstacle to the clinical application of large TE constructs is the poor perfusion, leading to cells in the central parts of the graft undergoing necrosis or ischemia due to inadequate support of oxygen and nutrients. This is a crucial problem for large and demanding vital organs like the liver, the kidney and heart. Considering the diffusion limit of oxygen is 150–200 μm, constructs greater than this size have to be properly vascularized in order to survive. Vessels growth *in vivo* is a very slow process, depending on the colonization of the construct by host cells that integrate and build a new capillary network. A further limitation is given by the development of vascular networks poorly organized, characterized by fluid leakage and hemorrhages and by the lack of proper anastomoses with the host vascular system. Importantly, TE might help overcome these problems, promoting the vascularization of big constructs.

### 3.1 Methods of vascularization

Two main strategies have been proposed to vascularize TE grafts (reviewed in Laschke and Menger, 2012, Rouwkema and Khademhosseini, 2016). The first one is the stimulation of neoangiogenesis *in situ* by host ECs and mural cells that colonize the graft post-implantation; this process relies on the expansion and growth of the host microvasculature within the graft (Laschke & Menger, 2012). As will be discussed in more details in the further session of this review, the scaffolds can be tailored to encourage the host cells invasion both by tuning its physical characteristics and by incorporating angiogenic factors (AFs).

The second strategy is based on the creation of a vascular network within the graft *in vitro,* pre-implantation. Once the graft is implanted, the pre-formed vascular network establishes connections with the host vasculature, process named inosculation (Laschke, Vollmar, & Menger, 2009). This process can be favored by the manufacture of materials guiding the formation of new tubular structures (see Section 3.3). Even if more elaborated, the establishment of pre-formed vasculature allows for a rapid connection to the host vascular network through newly formed anastomoses, guarantying a quick perfusion of the implant and better grafting chances.

For example, the transplantation of prevascularized collagen skin constructs in mice accelerated the formation of functional perfused vessels compared to non-vascularized grafts, corroborating the concept that the inosculation technique may favor the preservation of the TE graft, reducing the occurrence of ischemia (Tremblay, Hudon, Berthod, Germain, & Auger, 2005). Again, the prevascularization of fibrin constructs with human umbilical vein ECs (HUVECs) and fibroblasts prior to subcutaneous implantation in mice, promoted the perfusion of the grafts, accelerating the formation of anastomoses between the host and the engineered vascular network (Chen et al., 2009). Some literature reports underline that the formation of anastomoses to the host vasculature can be limited, as it happened for TE bone constructs vascularized with HUVECs prior to implantation into mice (Rouwkema, de Boer, & Van Blitterswijk, 2006). However, this latter might represent a limitation of the xenotransplant rather than a failure of the pre-vascularization method.

**Fig. 2:**
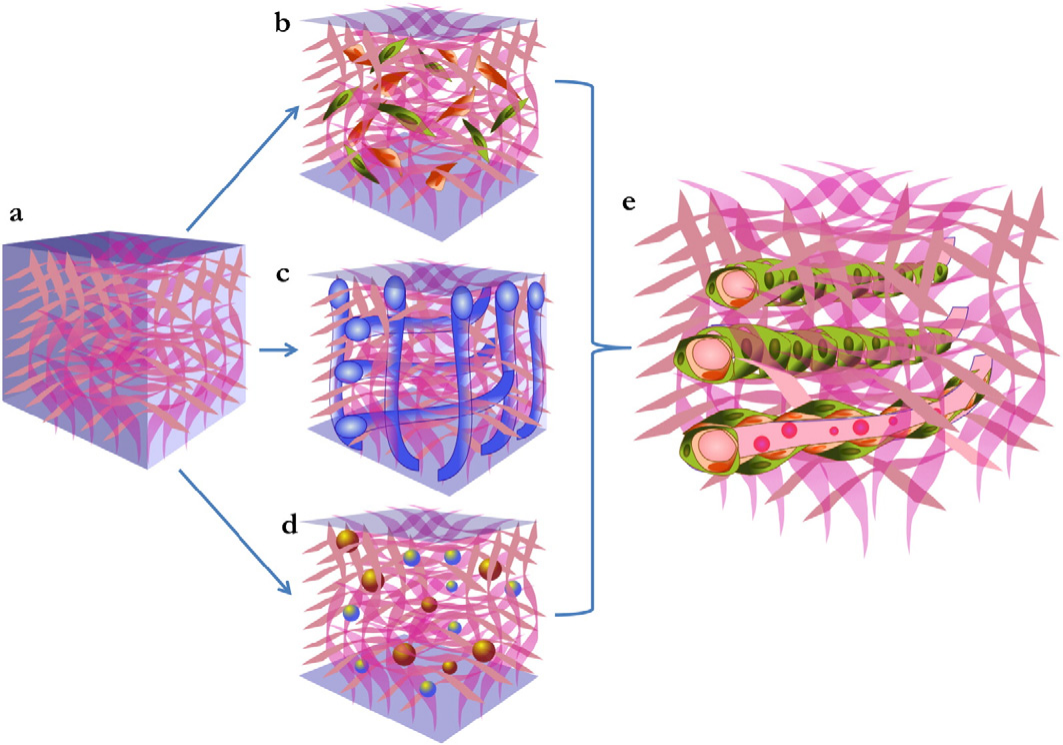
Strategies of vascularization of tissue-engineered grafts. Scaffolds used for TE applications (a) have to be uniformly vascularised in order to ensure the perfusion and survival of the inner parts. 3 main strategies have been proposed to promote this process: (b) combination of the material with cells able to support angiogenesis, (c) design of the scaffold structure reproducing vascular like-structures that will act as a guide for the angiogenic process, and (d) incorporation of angiogenic factors during the manufacture of the graft. The result should be a vascularised graft (e), in which new-formed vessels are mature and functionally competent.

As illustrated in Fig. 2, both vascularization strategies reported above can be optimized by the modification of several parameters: (1) the selection of the cellular candidate, (2) the design and fabrication of the scaffold structure and (3) the incorporation of AFs within the scaffold. In the following paragraphs we will briefly report on these points.

### 3.2 Use of perivascular cells to support angiogenesis

A combination of vascular ECs and perivascular/mural cells, such as fibroblasts, SMCs and pericytes, that physiologically form blood vessels and reside within the vascular niche, has largely been the preferred solution to promote the process of vascularization (Costa-Almeida, Granja, Soares, & Guerreiro, 2014).

The important role of PCs is supported by multiple literature reports. The implantation in mice of fibronectin/collagen I scaffolds seeded with HUVECs and MSCs enhanced the formation of a stable and functional vascular network; importantly, MSCs can differentiate into vascular mural cells, thus stabilizing the vessel structure (Koike et al., 2004). Sathy et al. used a mix of pericytes and ECs in order to optimize the vascularization of thick bone constructs, adopting a particular strategy based on the alternation of osteogenic and angiogenic layers, up to 400 μm in thickness. This peculiar organization allowed the Authors to generate functionally vascularized bone grafts after subcutaneous implantation in mice (Sathy et al., 2015). Also a study based on cell sheet technology for generation of bone constructs corroborated the importance of using PCs to achieve the formation of stable vascular networks. The integration of ECs, PCs and MSCs and the culture in osteogenic medium resulted in the generation of a well vascularized osteogenic construct (Mendes et al., 2012). Moreover, the seeding of 3D polyurethane scaffolds with human endothelial progenitor cells (EPCs) and MSCs yielded the formation of a vascularized graft presenting vascular structures made of cells positive for ECs markers and perivascular cells expressing antigens typical of pericytes, possibly derived from MSCs (Duttenhoefer et al., 2013).

### 3.3 Scaffold design and fabrication

As discussed above, the scaffold structure can influence the level of vascularization achieved both pre and post implantation. In particular, the porosity of the graft is a crucial parameter, since it determines the migration of cells inside the material, promoting blood vessels ingrowth. Pores bigger than 250 μm have been associated with a faster vascularization compared with smaller sizes (Druecke et al., 2004). Moreover, the connection between pores is essential to develop anastomoses between capillaries and build a functional vascular network.

Complex scaffold design prone to vascularization can be obtained by controlled manufacture processing such as electrospinning or microfluidic technologies.

Gafni et al. designed a biodegradable filamentous polymeric scaffold demonstrating this structure, combined with ECs, was able to guide the angiogenesis both *in vitro* and *in vivo.* Interestingly, the degradation of the polymer left space to a functional capillary network perfused by blood one month post-implantation in mice (Gafni et al., 2006). Again, the microfabrication of micro-channels scaffolds enhanced the vascularization of 2 mm thick alginate grafts by ECs both *in vitro* and *in vivo* (Zieber, Or, Ruvinov, & Cohen, 2014). A micromachining technology was employed to design complex branched silicon tubular networks able to act as a template for vascularization; integration of this system with hepatocytes and ECs allowed the Authors to engineer 3D liver tissue before implantation (Kaihara et al., 2000).

A recent study reports on the fabrication of 3D vascular structures using hydrogel electrospinning as templates for formation of new vasculature by ECs, pericytes and SMCs. Fibrin microfibers allowed the generation of complex and self-supporting vascular structures including not only the *tunica intima* made of ECs but also the *tunica media* composed of SMCs (Barreto-Ortiz et al., 2015).

The geometry of the engineered graft is also of paramount importance. A structure favoring the proper alignment of ECs in cord structures rather than the random formation of vascular networks can improve the functional properties of the vascularized graft *in vivo* (Zieber et al., 2014).

### 3.4 Incorporation of angiogenic factors

The embedding of AFs within the biomaterial is another strategy to promote the vascularization and the stabilization of newly formed vessels. Commonly employed AFs are PDGF, vascular endothelial growth factor (VEGF) and Angiopoietin 1 (Ang1), factors able to recruit ECs but also PCs (Chen et al., 2007, Hirschi et al., 2002). Additionally, the matrix can be loaded with factors stimulating the secretion of AFs by other cells, such as Sonic Hedgehog (SHH). SHH is able to stimulate the production of Ang1, Ang2 and VEGF in interstitial mesenchymal cells, boosting indirectly the angiogenesis in mice ischemic limbs (Pola et al., 2001). Also, the incorporation of VEGF and fibroblast growth factor-2 (FGF2) into a porous acellular scaffold increased the formation and maturation of new vessels 3 months after subcutaneous implantation in rats (Nillesen et al., 2007). Another study demonstrated that the functionalization of PLGA scaffolds pores with VEGF enhanced the vascularization *in vivo* after subcutaneous implantation. In the same study, the Authors provide a method of VEGF encapsulation within PLGA microspheres that improve the release of the GF over time, further boosting the process of angiogenesis (Ennett, Kaigler, & Mooney, 2006).

## 4 Use of perivascular cells for tissue engineering

**Fig. 3:**
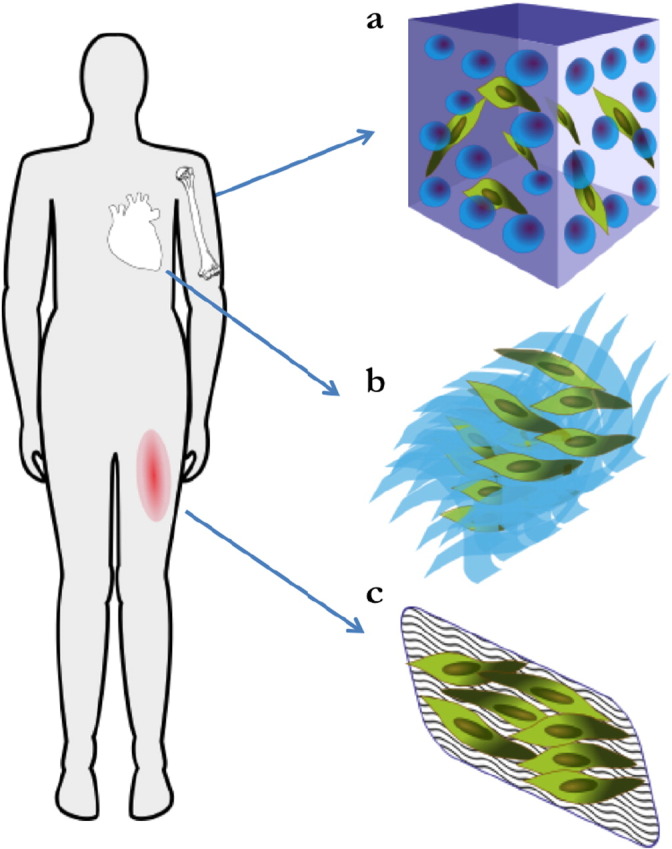
Application of perivascular cells for tissue engineering strategies of damaged tissues. The strategies of delivery of perivascular cells (PCs) for the repair of tissue defects are very divers and depend on the tissue/organ of interest: (a) bone reconstruction was achieved by incorporating PCs in 3D scaffolds; (b) heart patches obtained by stacking multilayers of PCs combined grown on matrix substrate were devised for myocardial infarction; (c) topical application of dermal patches containing PCs improved skin wound healing.

The amenability of PCs to TE applications has promoted their application for the repair of several tissues and organs. Below, we report on the engineering strategies adopted for the generation of vascular grafts and tissue-specific constructs aiming at the repair of the heart, skeletal muscle, bone and skin (Fig. 3).

### 4.1 Vascular grafts

#### 4.1.1 Limits of current grafting solutions

The progressive ageing of the world population and the rise of cardiovascular disease incidence have led to a growing number of bypass surgeries. The internal mammary artery and the saphenous vein are the surgeon's first choices; however while arterial grafts perform well both in the short and long term, about 50% of vein grafts become narrowed or occluded at 10 years, increasing to 85% after 15 years (Kannan, Salacinski, Butler, Hamilton, & Seifalian, 2005). The recurrence of the occlusion and the widespread presence of concurrent vascular pathologies lead to the compelling need of an artificial alternative for revascularization.

However, current clinically approved polymer-based grafts such as Dacron and ePFTE (polytetrafluoroethylene) have shown promising results as large vessel substitutes, but perform poorly for small diameter vessel bypass (< 6 mm) (Kannan et al., 2005).

The main complications observed are thrombosis and intima hyperplasia in the short/medium term and the development of stenosis and graft atherosclerosis in the long term (Yahagi et al., 2016), mainly due to compliance mismatch and thrombogenicity of the blood contacting surface.

The challenge of providing an adequate synthetic solution for small diameter vascular grafts has been taken up by TE. Many different solutions have been devised in terms of material science, ranging from decellularized vessels (Campagnolo et al., 2015, Tsai et al., 2012) to electrospun natural or synthetic fibers (as reviewed in Hasan et al., 2014).

Most commonly, tissue-engineered vascular grafts (TEVG) are seeded with ECs to mimic the natural blood-contacting surface, aiming at preventing the occurrence of blood clotting (Zhou et al., 2014). However, similarly to what discussed for the vascularization of TE scaffolds, the complete reconstruction of the EC lining is not sufficient to recapitulate the innate structure and physiological characteristics of the natural vessel, leading to poor mechanical characteristics and immature phenotype.

#### 4.1.2 Perivascular cells for vascular grafts

**Fig. 4:**
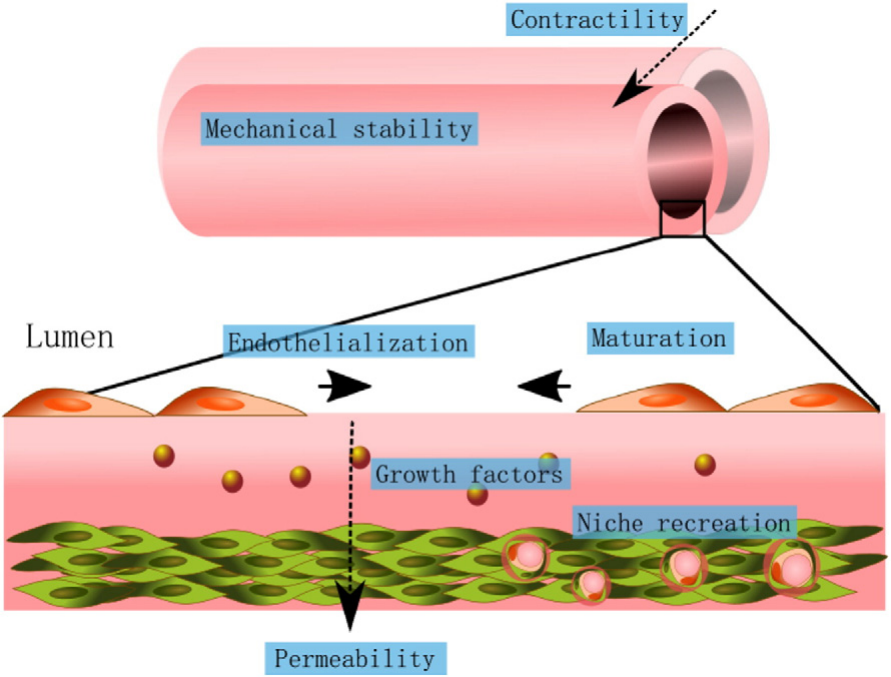
Role of perivascular cells in the generation of tissue engineered vascular grafts. The seeding of vascular grafts with perivascular cells increases their contractility and mechanical properties, regulating permeability. The release of growth factors by the perivascular cells regulates endothelialization and endothelial cell function. Additionally, perivascular cells contribute to the reconstitution of the perivascular niche, favoring the long-term graft success.

PCs are an obvious choice for vascular repopulation as they originate from the same anatomical district and can improve mechanical function, increase vessel contractility and reduce leaking. Furthermore, the natural crosstalk between PCs and ECs is re-established after implantation, leading to increased ECs proliferation and migration and promising a faster and more efficient re-endothelialization (Fig. 4).

The most common choice of PCs for TEVG is vascular SMCs, which are naturally located in the media layer of large vessel.

In a seminal Science study, Weinberg and Bell produced a tri-layer vascular graft incorporating 3 bovine primary cell populations: adventitial fibroblast, vascular SMCs and ECs in combination with a Dacron mesh, providing adequate support and an optimal barrier function (Weinberg & Bell, 1986). This initial proof-of-principle demonstrated the importance of recapitulating the vessel structure in the synthetic graft in order to incorporate all the physiological features of a natural vessel.

SMC phenotype and function are tightly regulated by their surrounding microenvironment and by their organization within the tissue (Rensen, Doevendans, & van Eys, 2007). For these reasons, the design of the graft material has to be informed by the knowledge of the biological environment in order to reduce complications derived by the over-proliferation of SMCs and improves the mechanical features of the synthetic graft. For example, it has been demonstrated in multiple studies that the rigidity of the matrix directly influences the phenotype of SMCs and the differentiation of progenitor cells (as reviewed in Liu, 2012).

Indeed, engineering approaches such as the production of grafts presenting aligned fibres (Agrawal et al., 2015) or the harnessing of the differentiative effect of the substrate stiffness (Floren et al., 2016) were able to produce functionally superior vascular grafts.

For example, to improve the mechanical properties of the grafts, SMCs were seeded circumferentially around a central tubular mould (Agrawal et al., 2015, L'Heureux et al., 1993) or on highly organized collagen fibres (Barocas, Girton, & Tranquillo, 1998). The addition of cyclic strain (Seliktar, Black, Vito, & Nerem, 2000) alone or in combination with GFs (Stegemann & Nerem, 2003) also determined an improvement in the mechanical features of the graft and provided an optimal orientation of the medial cells. Additionally, the material design can be implemented by incorporating a matrix providing slow release of an anti-proliferative drug to control the SMC phenotype (Wu, Liu, Chen, Yang, & Zhu, 2016).

Despite the success of the use of mature SMCs and ECs in preclinical models, their use in the clinical setting is hampered by the difficulty in sourcing of patients' autologous cells due to the early SMCs senescence (McKee et al., 2003) and the lack of elastin production by mature cells, leading to insufficient compliance (Long & Tranquillo, 2003).

This observation led to the proliferation of protocols aimed at obtaining pure populations of vascular cells from embryonic stem cells (ES) and iPS precursors as well as the isolation of SMC precursors from bone marrow derived SMCs (Swaminathan, Sivaraman, Moore, Zborowski, & Ramamurthi, 2016).

The application of these technologies is still limited by the lack of a simple and safe protocol for differentiation, leading to lengthy and costly reprogramming and scale up procedures mostly based on viral infections and xeno supplements (Kusuma and Gerecht, 2016, Patsch et al., 2015).

#### 4.1.3 Pericyte potential for vascular graft engineering

Pericytes are an interesting choice for vascular graft repopulation as they present progenitor cell characteristics but their differentiation capacity and proliferation is restricted, reducing the risk of tumorigenesis. Human pericytes isolation, characterisation and expansion at clinical level are relatively recent but it has already given rise to several important preclinical studies (Avolio et al., 2015b, Campagnolo et al., 2010, Carrabba et al., 2016, Chen et al., 2015, Gubernator et al., 2015, Katare et al., 2011). Their use for vascular graft repopulation is still limited but the studies published to date indicate a very promising route of investigation.

Human SkPs have been used to repopulate a poly(ester-urethane)urea 1.3 mm diameter scaffold and cultured in a bioreactor before implantation in rats. The aims of this report were limited to assessing the feasibility and efficiency of uniform cell seeding using a new vacuum-based technique and the *in vivo* performance of the resulting graft in a xeno-transplant model. However, the results showed an impressive performance of the seeded grafts in terms of patency and an active remodelling and rapid invasion from the host cells, with formation of medial and intimal layers (He et al., 2010). The study of the mechanisms involved in the remodelling and the improved migration of the host cells into the graft as a consequence of the pericyte seeding warrants further investigation and harbours the potential for harnessing the unique characteristics of pericytes into the engineering of better forming vascular grafts. Our unpublished data show how the synthetic graft material can be modified to incorporate bioactive peptides capable of retaining and releasing SVP-produced growth factors. This design innovation combined with spatially organized EC-specific adhesion peptides greatly improved the endothelial coverage of the graft (Campagnolo et al., 2016).

As shown by previous *in vitro* studies, pericytes possess the innate ability to increase ECs proliferation/survival and migration. In a proof of concept paper, Chong et al. utilized the readily available UCPCs to engineer an *in vitro* pseudo-vessel where they seeded pericytes on a hydrogel-modified PCL film and then tethered EPCs on the other surface by functionalizing it with anti-CD34 antibody. This study demonstrates that the pro-angiogenic potential of pericytes can be harnessed for recapitulating the vessel structure and that a bioengineering approach can be applied to design a complex multicellular *in vitro* model to study cell mechanisms and interactions at basic level and to elucidate pathological processes (Chong, Chan, Choolani, Lee, & Teoh, 2009).

Pericytes' therapeutic capacity lays in their ability to release a large variety of GFs and cytokines and in the context of TEVG these substances contributes to the recruitment of vascular cells from the neighbouring vessel. The remodelling determined by the invasion of the host cells and in particular of the vascular resident progenitor cells is fundamental for the digestion of bioresorbable vascular graft materials and the reduction of compliance mismatching (Chen et al., 2013, Pasquinelli et al., 2007). Importantly, the release of paracrine substances such as TGFβ, in combination with the expression of the major histocompatibility complex (MHC) class I on the cell surfaces is also responsible for the reported immunomodulatory effect of pericytes (Domev, Milkov, Itskovitz-Eldor, & Dar, 2014). The pericyte's ability to elude the immune system and modulate the inflammatory response opens the way for the creation of allogeneic and off-the-shelf TEVG incorporating a perivascular cellular component, reducing the costs of production and scaling up the availability. This is particularly compelling given the recent findings elucidating the epigenetic mechanisms underlying the divergent success of the expansion of different cell lines and therefore suggesting a rapid way of assessing the suitability of selected cell lines for scale-up (Gubernator et al., 2015).

Ultimately, the use of pericytes in TEVG might help recreating the adventitial *vasa vasorum* niche, which is fundamental for the healthy functionality of the large vessels and therefore contributing to the successful integration of the graft (Guillemette et al., 2010, Orrico et al., 2010).

### 4.2 Skeletal and cardiac muscle grafts

Heart failure, particularly MI, is one of the leading causes of morbidity and mortality in the world. Stem cell transplantation therapy has emerged as a popular strategy to treat heart dysfunction. Direct injection of cell suspensions using catheters or open chest heart surgery is the most common method employed. Despite the increasing number of clinical trials carried out using direct injection approach, no cell therapy has been shown to be effective in a conclusive manner (Sanganalmath & Bolli, 2013). TE offers an alternative approach for cell delivery that can improve cell retention, survival and communication with the neighboring native tissue. A number of studies have explored the use of this approach to deliver pericytes into cardiac (Avolio et al., 2015a, Wendel et al., 2015) and skeletal (Dellavalle et al., 2007, Fuoco et al., 2014, Rossi et al., 2011) muscles. Wendel et al. produced a cardiac patch by enmeshing human BPs and human iPS derived cardiomyocytes into a fibrin gel (Wendel et al., 2015). Once transplanted onto the infarcted myocardium of a rat, this pericytes/cardiomyocytes patch survived, improved cardiac function and reduced infarct size. We have recently isolated and characterized human neonatal CPs and shown that, when engrafted into a decellularized xenograft (CorMatrix® ECM®), the pericyte-engineered tissue offers a new option of autologous cardiovascular grafts for corrective surgery of congenital heart defects (Avolio, Rodriguez-Arabaolaza, et al., 2015).

Additionally, the myogenic capacity and the pro-angiogenic ability of SkPs were harnessed by organizing them in a poly(ethylene glycol) PEG hydrogel-based construct for the repair of ischemic muscle (Fuoco et al., 2014). The presence of the matrix significantly increased the effect of the cells by recreating the niche necessary for the survival of the transplanted cells. A similar approach has been previously applied to satellite cells, a distinct skeletal muscle progenitor cell population (Dellavalle et al., 2007), by including them in a hyaluronan-based hydrogel (Rossi et al., 2011).

### 4.3 Bone regeneration

In orthopedics and related specialities, bone loss and repair are clinically and economically very important. As the population continues to age, skeletal diseases' costs are expected to rise. TE has the promise to improve bone regeneration (formation) in skeletal diseases. Bone marrow mesenchymal stem cells (BMSCs) are the main source of stem cell for bone regeneration (Deans & Moseley, 2000). However, when autogenous BMSCs are in short supply, purified perivascular cells from adipose tissue could offer an attractive alternative. A recent study has shown that, when human adipose tissue PCs are engrafted into a scaffold of morselized cortical and cancellous bone chips mixed with sodium hyaluronate and implanted in a rat model of spinal fusion, these adipose tissue PCs differentiated into osteoblasts and osteocytes (Chung et al., 2014). Additionally they triggered new bone formation of host origin, most likely, through direct and paracrine mechanisms.

Scaffolds containing a high dose of human adipose tissue PCs combined with the osteogenic protein NELL-1 have been shown to increase spinal fusion in osteoporotic rats. In contrast, regular doses of adipose tissue PCs or NELL-1 achieved only modest fusion rates suggesting that the combination synergistically enhances spinal fusion in osteoporotic rats. This study confirmed the potential of adipose tissue PCs and NELL-1 as a novel therapy for osteoporotic patients (Lee et al., 2015).

Furthermore, human adipose tissue PCs seeded onto a PLGA scaffold were shown to increase healing of mouse critical-size calvarial defects within 2 weeks of delivery (James et al., 2012). This is yet another example showing human PCs potential in skeletal regenerative medicine.

In addition to adipose tissue, UCPCs showed great potential in bone TE. Indeed when delivered together with a collagen sponge scaffold or an alginate gel, UCPCs significantly increased bone repair in a calvarial osteotomy model after 4 weeks of transplantation (Kajiyama et al., 2015). Similarly human CD146 + UCPCs seeded onto gelfoam-alginate 3D scaffold and transplanted subcutaneously into immunodeficient mice, resulted in the formation of bone matrix with embedded osteocytes of donor origin (Tsang et al., 2013).

### 4.4 Dermal tissue engineering

The current treatments of skin diseases fail to result in optimal healing. This is in part due to their inability to completely restore the function and structure of the dermis. Dermal tissue engineering has revolved around using different cell types for the treatment of cutaneous wounds by direct injection or scaffold-based delivery system. Human UCPCs have recently been shown to have great potential for the treatment of skin wounds (Zebardast, Lickorish, & Davies, 2010). Compared to human BMSCs, already used in clinical treatment of skin defects, UCPCs exhibited a higher proliferative rate. Furthermore, UCPCs promoted healing of full thickness murine skin defects when delivered in the wound site *via* a polymerized fibrin patch. This study demonstrated that UCPCs represent a great cell source for dermal tissue engineering and dermal repair.

## 5 Future directions

PCs have contributed substantially to the shaping of the TE field. Their use has been extensive, in particular for the vascularization of constructs and for repopulation of vascular grafts. Mature PCs, such as SMCs, have found wide application in the past. However their laborious and patient-specific sourcing, limited expansion capacity and the lack of standardized isolation protocols hinder their therapeutic application. The recent emergence of human pericytes led to the development of new routes for the preparation of off-the-shelf engineered tissues, thanks to their immunomodulatory effect and high proliferative capacity.

Despite some interesting publications in the recent years, the exploration of the potential of pericytes for TE still requires further research to establish the effect of the scaffold materials on their function and phenotype and to study in details the mechanisms involved in the remodeling post-implantation. Importantly, while the safety of pericyte transplantation has been evaluated in terms of immunological response and no neoplastic behavior has been reported *in vivo* or *in vitro*, long term studies in immune deficient models is warranted to exclude any potential for tumorigenesis.

Furthermore, the design of materials for TE needs to be developed to enhance and improve the cellular function and include features to direct cell differentiation and behaviour. In this way, the biological function of PCs will inform the design and development of advanced healthcare materials.

## Conflict of interest statement

The authors declare that there are no conflicts of interest.

## Funding acknowledgements

Authors are supported by: the British Heart Foundation PG/15/32/31398 “Neonatal Cardiac Pericytes engineered grafts for correction of congenital heart defects” (EA); the Heart Research UK RG2639 “Unravelling the molecular mechanisms of human adventitial pericytes for clinical translation” (VVA); The Sir Jules Thorn Award for Biomedical Research and the Enid Linder Foundation (MTG). Also, this work was funded by the BHF Centre of Regenerative Medicine (RM/13/2/30158).

## Figures and Tables

**Table 1:** Comparison of marker expression between mesenchymal stem cells (MSCs), vascular smooth muscle cells (SMCs), pericytes and endothelial cells (ECs).

Cell type	Source	Phenotype	References
MSCs	Bone marrow, adipose tissue, peripheral blood, other tissues	CD44 +/CD90 +/CD105 +/CD73 +/CD146 +/CD34 −/CD45 −/CD14 −	Hass et al. (2011)
SMCs	Arteries, veins	Synthetic: vimentin +/Smemb +/tropomyosin +/CRBP1 + Contractile: α-SMA +/Transgelin +/SMMHC +	Wanjare, Kusuma, and Gerecht (2014)
Pericytes and pericyte-associated cells	Capillaries/microvessels from various tissues	NG2 +/PDGFRβ +/vimentin + CD146 + or −/CD34 + or − CD44 +/CD90 +/CD105 +/CD73 +/CD31 −/CD45 −/CD56 −	Campagnolo et al. (2010) Chen et al., (2015) Avolio, Meloni, et al. (2015)
ECs	Vascular intima	CD31 +/CD144 +/vWF +/CD34 +/CD45 −	Bompais et al. (2004)

**Table 2:** Characteristics of pericytes and pericyte-associated isolated from different sources.

Pericytes and source	Strategy of isolation	Phenotype in culture	Characteristics/functions	References
Saphenous vein pericytes (SVPs), from saphenous vein	CD34 +/CD31 − magnetic bead selection	*Positive:* NG2, PDGFRβ, CD44, CD90, CD105, CD73, VIMENTIN *Negative:* CD146, CD45, CD31	Stabilization/control, blood vessel permeability, blood pressure, vasculogenesis, angiogenesis; Physiological/pathological repair process.	Campagnolo et al. (2010)
Cardiac pericyte-associated (CPs), from neonatal atrium/ventricle	CD34 +/CD31 − magnetic bead selection	*Positive:* NG2, PDGFRβ, CD44, CD90, CD105, CD73 *Negative:* CD146, CD45, CD31	Angiogenesis, ECM protein secretion.	Avolio, Rodriguez-Arabaolaza, et al. (2015)
Myocardial pericytes (MPs), from fetal/adult hearts	CD146 +/CD34 −/CD45 −/CD56 −/CD117 − Fluorescent activated cell sorting	*Positive:* NG2, PDGFRβ, CD44, CD90, CD105, CD73, VIMENTIN, CD146 *Negative:* CD34, CD45, CD31	Angiogenesis; vascular permeability control; blood flow regulation; trophic functions; ECM protein secretion.	Chen et al. (2015)
Skeletal muscle pericytes (SkPs), from Skeletal muscle	CD146high/CD34 − Fluorescent activated cell sorting	*Positive:* CD146 *Negative:* CD34, CD45, CD144, CD56, CD31	Myogenic potential; Role in muscle ontogeny and regeneration; Promote assembling of new vasculature in skeletal muscle.	Crisan et al. (2008)
Brain pericytes (BPs), from brain microvasculature	Cloning and morphology	*Positive:* PDGFRβ, α-SMA, 3G5, RGS5, MHC I-II *Negative:* CD45, vWF	Control of BBB integrity Regulation of microvessel architecture; ECM protein secretion; Regulation of capillary diameter and blood flow; Phagocytic functions.	Bouchard et al. (1997) Winkler, Bell, and Zlokovic (2011)
Liver pericytes (LPs) from hepatic tissue	Density gradient or Fluorescent sorting based on endogenous retinol or Liver explant outgrowth	*Positive:* α*-*SMA, NG2, DESMIN, GFAP	Retinol transport and storage; TGFβ-dependent ECM regulation; Angiogenesis and sinusoidal remodelling.	Friedman and Roll (1987); Matsuura et al. (1989) Blazejewski et al. (1995) Yokoi et al. (1984) Friedman (2008)
Dental pulp pericytes (DPPs), from dental pulp	STRO1 + magnetic bead selection	*Positive:* STRO-1, CD146, 3G5, α-SMA *Negative:* vWF	High proliferative potential; Regeneration of mineralized structure as bone and dentin; Support hematopoiesis.	Shi and Gronthos (2003) Alliot-Licht et al. (2005)

## References

[bb0005] Agrawal A., Lee B.H., Irvine S.A., An J., Bhuthalingam R., Singh V., Venkatraman S.S. (2015). Smooth muscle cell alignment and phenotype control by melt spun polycaprolactone fibers for seeding of tissue engineered blood vessels. International Journal of Biomaterials.

[bb0010] Alliot-Licht B., Bluteau G., Magne D., Lopez-Cazaux S., Lieubeau B., Daculsi G., Guicheux J. (2005). Dexamethasone stimulates differentiation of odontoblast-like cells in human dental pulp cultures. Cell and Tissue Research.

[bb0015] Andreas K., Sittinger M., Ringe J. (2014). Toward in situ tissue engineering: Chemokine-guided stem cell recruitment. Trends in Biotechnology.

[bb0020] Avolio E., Meloni M., Spencer H.L., Riu F., Katare R., Mangialardi G., Madeddu P. (2015). Combined intramyocardial delivery of human pericytes and cardiac stem cells additively improves the healing of mouse infarcted hearts through stimulation of vascular and muscular repair. Circulation Research.

[bb0025] Avolio E., Rodriguez-Arabaolaza I., Spencer H.L., Riu F., Mangialardi G., Slater S.C., Madeddu P. (2015). Expansion and characterization of neonatal cardiac pericytes provides a novel cellular option for tissue engineering in congenital heart disease. Journal of the American Heart Association.

[bb0030] Barocas V.H., Girton T.S., Tranquillo R.T. (1998). Engineered alignment in media equivalents: Magnetic prealignment and mandrel compaction. Journal of Biomechanical Engineering.

[bb0035] Barreto-Ortiz S.F., Fradkin J., Eoh J., Trivero J., Davenport M., Ginn B., Gerecht S. (2015). Fabrication of 3-dimensional multicellular microvascular structures. The FASEB Journal.

[bb0040] Blazejewski S., Preaux A.M., Mallat A., Brocheriou I., Mavier P., Dhumeaux D., Rosenbaum J. (1995). Human myofibroblastlike cells obtained by outgrowth are representative of the fibrogenic cells in the liver. Hepatology.

[bb0045] Bompais H., Chagraoui J., Canron X., Crisan M., Liu X.H., Anjo A., Uzan G. (2004). Human endothelial cells derived from circulating progenitors display specific functional properties compared with mature vessel wall endothelial cells. Blood.

[bb0050] Bouchard B.A., Shatos M.A., Tracy P.B. (1997). Human brain pericytes differentially regulate expression of procoagulant enzyme complexes comprising the extrinsic pathway of blood coagulation. Arteriosclerosis, Thrombosis, and Vascular Biology.

[bb0055] Campagnolo P., Cesselli D., Al Haj Zen A., Beltrami A.P., Krankel N., Katare R., Madeddu P. (2010). Human adult vena saphena contains perivascular progenitor cells endowed with clonogenic and proangiogenic potential. Circulation.

[bb0060] Campagnolo P., Gormley A.J., Chow L.W., Guex A.G., Parmar P.A., Puetzer J., Stevens M.M. (2016). Pericyte-coated dual peptide scaffold with improved endothelialization for vascular graft tissue engineering. Advanced Healthcare Materials.

[bb0065] Campagnolo P., Tsai T.N., Hong X., Kirton J.P., So P.W., Margariti A., Xu Q. (2015). c-Kit + progenitors generate vascular cells for tissue-engineered grafts through modulation of the Wnt/Klf4 pathway. Biomaterials.

[bb0070] Carrabba M., De Maria C., Oikawa A., Reni C., Rodriguez-Arabaolaza I., Spencer H., Vozzi G. (2016). Design, fabrication and perivascular implantation of bioactive scaffolds engineered with human adventitial progenitor cells for stimulation of arteriogenesis in peripheral ischemia. Biofabrication.

[bb0075] Chen X., Aledia A.S., Ghajar C.M., Griffith C.K., Putnam A.J., Hughes C.C., George S.C. (2009). Prevascularization of a fibrin-based tissue construct accelerates the formation of functional anastomosis with host vasculature. Tissue Engineering. Part A.

[bb0080] Chen W.C., Baily J.E., Corselli M., Diaz M.E., Sun B., Xiang G., Peault B. (2015). Human myocardial pericytes: multipotent mesodermal precursors exhibiting cardiac specificity. Stem Cells.

[bb0085] Chen R.R., Silva E.A., Yuen W.W., Mooney D.J. (2007). Spatio-temporal VEGF and PDGF delivery patterns blood vessel formation and maturation. Pharmaceutical Research.

[bb0090] Chen Y., Wong M.M., Campagnolo P., Simpson R., Winkler B., Margariti A., Xu Q. (2013). Adventitial stem cells in vein grafts display multilineage potential that contributes to neointimal formation. Arteriosclerosis, Thrombosis, and Vascular Biology.

[bb0095] Chong M.S., Chan J., Choolani M., Lee C.N., Teoh S.H. (2009). Development of cell-selective films for layered co-culturing of vascular progenitor cells. Biomaterials.

[bb0100] Chung C.G., James A.W., Asatrian G., Chang L., Nguyen A., Le K., Soo C. (2014). Human perivascular stem cell-based bone graft substitute induces rat spinal fusion. Stem Cells Translational Medicine.

[bb0105] Corselli M., Chin C.J., Parekh C., Sahaghian A., Wang W., Ge S., Peault B. (2013). Perivascular support of human hematopoietic stem/progenitor cells. Blood.

[bb0110] Costa-Almeida R., Granja P.L., Soares R., Guerreiro S.G. (2014). Cellular strategies to promote vascularisation in tissue engineering applications. European Cells & Materials.

[bb0115] Covas D.T., Panepucci R.A., Fontes A.M., Silva W.A., Orellana M.D., Freitas M.C., Zago M.A. (2008). Multipotent mesenchymal stromal cells obtained from diverse human tissues share functional properties and gene-expression profile with CD146 + perivascular cells and fibroblasts. Experimental Hematology.

[bb0120] Crisan M., Corselli M., Chen W.C., Peault B. (2012). Perivascular cells for regenerative medicine. Journal of Cellular and Molecular Medicine.

[bb0125] Crisan M., Yap S., Casteilla L., Chen C.W., Corselli M., Park T.S., Peault B. (2008). A perivascular origin for mesenchymal stem cells in multiple human organs. Cell Stem Cell.

[bb0130] Dar A., Itskovitz-Eldor J. (2015). Therapeutic potential of perivascular cells from human pluripotent stem cells. Journal of Tissue Engineering and Regenerative Medicine.

[bb0135] Dar A., Domev H., Ben-Yosef O., Tzukerman M., Zeevi-Levin N., Novak A., Itskovitz-Eldor J. (2012). Multipotent vasculogenic pericytes from human pluripotent stem cells promote recovery of murine ischemic limb. Circulation.

[bb0140] Deans R.J., Moseley A.B. (2000). Mesenchymal stem cells: Biology and potential clinical uses. Experimental Hematology.

[bb0145] Dellavalle A., Sampaolesi M., Tonlorenzi R., Tagliafico E., Sacchetti B., Perani L., Cossu G. (2007). Pericytes of human skeletal muscle are myogenic precursors distinct from satellite cells. Nature Cell Biology.

[bb0150] Domev H., Milkov I., Itskovitz-Eldor J., Dar A. (2014). Immunoevasive pericytes from human pluripotent stem cells preferentially modulate induction of allogeneic regulatory T cells. Stem Cells Translational Medicine.

[bb0155] Druecke D., Langer S., Lamme E., Pieper J., Ugarkovic M., Steinau H.U., Homann H.H. (2004). Neovascularization of poly(ether ester) block-copolymer scaffolds in vivo: Long-term investigations using intravital fluorescent microscopy. Journal of Biomedical Materials Research. Part A.

[bb0160] Duttenhoefer F., Lara de Freitas R., Meury T., Loibl M., Benneker L.M., Richards R.G., Verrier S. (2013). 3D scaffolds co-seeded with human endothelial progenitor and mesenchymal stem cells: Evidence of prevascularisation within 7 days. European Cells & Materials.

[bb0165] Ennett A.B., Kaigler D., Mooney D.J. (2006). Temporally regulated delivery of VEGF in vitro and in vivo. Journal of Biomedical Materials Research. Part A.

[bb0170] Floren M., Bonani W., Dharmarajan A., Motta A., Migliaresi C., Tan W. (2016). Human mesenchymal stem cells cultured on silk hydrogels with variable stiffness and growth factor differentiate into mature smooth muscle cell phenotype. Acta Biomaterialia.

[bb0175] Friedman S.L. (2008). Hepatic stellate cells: Protean, multifunctional, and enigmatic cells of the liver. Physiological Reviews.

[bb0180] Friedman S.L., Roll F.J. (1987). Isolation and culture of hepatic lipocytes, Kupffer cells, and sinusoidal endothelial cells by density gradient centrifugation with Stractan. Analytical Biochemistry.

[bb0185] Fuoco C., Sangalli E., Vono R., Testa S., Sacchetti B., Latronico M.V., Gargioli C. (2014). 3D hydrogel environment rejuvenates aged pericytes for skeletal muscle tissue engineering. Frontiers in Physiology.

[bb0190] Gafni Y., Zilberman Y., Ophir Z., Abramovitch R., Jaffe M., Gazit Z., Gazit D. (2006). Design of a filamentous polymeric scaffold for in vivo guided angiogenesis. Tissue Engineering.

[bb0195] Gokcinar-Yagci B., Uckan-Cetinkaya D., Celebi-Saltik B. (2015). Pericytes: Properties, functions and applications in tissue engineering. Stem Cell Reviews.

[bb0200] Gubernator M., Slater S.C., Spencer H.L., Spiteri I., Sottoriva A., Riu F., Madeddu P. (2015). Epigenetic profile of human adventitial progenitor cells correlates with therapeutic outcomes in a mouse model of limb ischemia. Arteriosclerosis, Thrombosis, and Vascular Biology.

[bb0205] Guillemette M.D., Gauvin R., Perron C., Labbe R., Germain L., Auger F.A. (2010). Tissue-engineered vascular adventitia with vasa vasorum improves graft integration and vascularization through inosculation. Tissue Engineering. Part A.

[bb0210] Hasan A., Memic A., Annabi N., Hossain M., Paul A., Dokmeci M.R., Khademhosseini A. (2014). Electrospun scaffolds for tissue engineering of vascular grafts. Acta Biomaterialia.

[bb0215] Hass R., Kasper C., Bohm S., Jacobs R. (2011). Different populations and sources of human mesenchymal stem cells (MSC): A comparison of adult and neonatal tissue-derived MSC. Cell Communication and Signaling: CCS.

[bb0220] He W., Nieponice A., Soletti L., Hong Y., Gharaibeh B., Crisan M., Vorp D.A. (2010). Pericyte-based human tissue engineered vascular grafts. Biomaterials.

[bb0225] Hirschi K.K., Skalak T.C., Peirce S.M., Little C.D. (2002). Vascular assembly in natural and engineered tissues. Annals of the New York Academy of Sciences.

[bb0230] James A.W., Zara J.N., Corselli M., Askarinam A., Zhou A.M., Hourfar A., Soo C. (2012). An abundant perivascular source of stem cells for bone tissue engineering. Stem Cells Translational Medicine.

[bb0235] Kaihara S., Borenstein J., Koka R., Lalan S., Ochoa E.R., Ravens M., Vacanti J.P. (2000). Silicon micromachining to tissue engineer branched vascular channels for liver fabrication. Tissue Engineering.

[bb0240] Kajiyama S., Ujiie Y., Nishikawa S., Inoue K., Shirakawa S., Hanada N., Gomi K. (2015). Bone formation by human umbilical cord perivascular cells. Journal of Biomedical Materials Research. Part A.

[bb0245] Kannan R.Y., Salacinski H.J., Butler P.E., Hamilton G., Seifalian A.M. (2005). Current status of prosthetic bypass grafts: A review. Journal of Biomedical Materials Research. Part B, Applied Biomaterials.

[bb0250] Katare R., Riu F., Mitchell K., Gubernator M., Campagnolo P., Cui Y., Madeddu P. (2011). Transplantation of human pericyte progenitor cells improves the repair of infarcted heart through activation of an angiogenic program involving micro-RNA-132. Circulation Research.

[bb0255] Keane T.J., Badylak S.F. (2014). Biomaterials for tissue engineering applications. Seminars in Pediatric Surgery.

[bb0260] Koike N., Fukumura D., Gralla O., Au P., Schechner J.S., Jain R.K. (2004). Tissue engineering: Creation of long-lasting blood vessels. Nature.

[bb0265] Kovacic J.C., Boehm M. (2009). Resident vascular progenitor cells: An emerging role for non-terminally differentiated vessel-resident cells in vascular biology. Stem Cell Research.

[bb0270] Kusuma S., Gerecht S. (2016). Derivation of endothelial cells and pericytes from human pluripotent stem cells. Methods in Molecular Biology.

[bb0275] Kusuma S., Facklam A., Gerecht S. (2015). Characterizing human pluripotent-stem-cell-derived vascular cells for tissue engineering applications. Stem Cells and Development.

[bb0280] Kusuma S., Macklin B., Gerecht S. (2014). Derivation and network formation of vascular cells from human pluripotent stem cells. Methods in Molecular Biology.

[bb0285] Laschke M.W., Menger M.D. (2012). Vascularization in tissue engineering: Angiogenesis versus inosculation. European Surgical Research.

[bb0290] Laschke M.W., Vollmar B., Menger M.D. (2009). Inosculation: Connecting the life-sustaining pipelines. Tissue Engineering. Part B, Reviews.

[bb0295] Lee E.J., Kasper F.K., Mikos A.G. (2014). Biomaterials for tissue engineering. Annals of Biomedical Engineering.

[bb0300] Lee S., Zhang X., Shen J., James A.W., Chung C.G., Hardy R., Soo C. (2015). Brief report: Human perivascular stem cells and Nel-like protein-1 synergistically enhance spinal fusion in osteoporotic rats. Stem Cells.

[bb0305] L'Heureux N., Germain L., Labbe R., Auger F.A. (1993). In vitro construction of a human blood vessel from cultured vascular cells: A morphologic study. Journal of Vascular Surgery.

[bb0310] Li Y., Meng H., Liu Y., Lee B.P. (2015). Fibrin gel as an injectable biodegradable scaffold and cell carrier for tissue engineering. ScientificWorldJournal.

[bb0315] Liu W.F. (2012). Mechanical regulation of cellular phenotype: Implications for vascular tissue regeneration. Cardiovascular Research.

[bb0320] Long J.L., Tranquillo R.T. (2003). Elastic fiber production in cardiovascular tissue-equivalents. Matrix Biology.

[bb0325] Matsuura T., Nagamori S., Fujise K., Hasumura S., Homma S., Sujino H., Hirosawa K. (1989). Retinol transport in cultured fat-storing cells of rat liver. Quantitative analysis by anchored cell analysis and sorting system. Laboratory Investigation.

[bb0330] Mazza G., Rombouts K., Rennie Hall A., Urbani L., Vinh Luong T., Al-Akkad W., Pinzani M. (2015). Decellularized human liver as a natural 3D-scaffold for liver bioengineering and transplantation. Scientific Reports.

[bb0335] McKee J.A., Banik S.S., Boyer M.J., Hamad N.M., Lawson J.H., Niklason L.E., Counter C.M. (2003). Human arteries engineered in vitro. EMBO Reports.

[bb0340] Mendes L.F., Pirraco R.P., Szymczyk W., Frias A.M., Santos T.C., Reis R.L., Marques A.P. (2012). Perivascular-like cells contribute to the stability of the vascular network of osteogenic tissue formed from cell sheet-based constructs. PLoS One.

[bb0345] Miranville A., Heeschen C., Sengenes C., Curat C.A., Busse R., Bouloumie A. (2004). Improvement of postnatal neovascularization by human adipose tissue-derived stem cells. Circulation.

[bb0350] Naderi-Meshkin H., Bahrami A.R., Bidkhori H.R., Mirahmadi M., Ahmadiankia N. (2015). Strategies to improve homing of mesenchymal stem cells for greater efficacy in stem cell therapy. Cell Biology International.

[bb0355] Nillesen S.T., Geutjes P.J., Wismans R., Schalkwijk J., Daamen W.F., van Kuppevelt T.H. (2007). Increased angiogenesis and blood vessel maturation in acellular collagen-heparin scaffolds containing both FGF2 and VEGF. Biomaterials.

[bb0360] Orlova V.V., Drabsch Y., Freund C., Petrus-Reurer S., van den Hil F.E., Muenthaisong S., Mummery C.L. (2014). Functionality of endothelial cells and pericytes from human pluripotent stem cells demonstrated in cultured vascular plexus and zebrafish xenografts. Arteriosclerosis, Thrombosis, and Vascular Biology.

[bb0365] Orlova V.V., van den Hil F.E., Petrus-Reurer S., Drabsch Y., Ten Dijke P., Mummery C.L. (2014). Generation, expansion and functional analysis of endothelial cells and pericytes derived from human pluripotent stem cells. Nature Protocols.

[bb0370] Orrico C., Pasquinelli G., Foroni L., Muscara D., Tazzari P.L., Ricci F., Stella A. (2010). Dysfunctional vasa vasorum in diabetic peripheral artery obstructive disease with critical lower limb ischaemia. European Journal of Vascular and Endovascular Surgery.

[bb0375] Owens G.K., Kumar M.S., Wamhoff B.R. (2004). Molecular regulation of vascular smooth muscle cell differentiation in development and disease. Physiological Reviews.

[bb0380] Park T.S., Gavina M., Chen C.W., Sun B., Teng P.N., Huard J., Peault B. (2011). Placental perivascular cells for human muscle regeneration. Stem Cells and Development.

[bb0385] Pasquinelli G., Tazzari P.L., Vaselli C., Foroni L., Buzzi M., Storci G., Conte R. (2007). Thoracic aortas from multiorgan donors are suitable for obtaining resident angiogenic mesenchymal stromal cells. Stem Cells.

[bb0390] Patsch C., Challet-Meylan L., Thoma E.C., Urich E., Heckel T., O'Sullivan J.F., Cowan C.A. (2015). Generation of vascular endothelial and smooth muscle cells from human pluripotent stem cells. Nature Cell Biology.

[bb0395] Pola R., Ling L.E., Silver M., Corbley M.J., Kearney M., Blake Pepinsky R., Isner J.M. (2001). The morphogen Sonic hedgehog is an indirect angiogenic agent upregulating two families of angiogenic growth factors. Nature Medicine.

[bb0400] Rensen S.S., Doevendans P.A., van Eys G.J. (2007). Regulation and characteristics of vascular smooth muscle cell phenotypic diversity. Netherlands Heart Journal.

[bb0405] Rossi C.A., Flaibani M., Blaauw B., Pozzobon M., Figallo E., Reggiani C., De Coppi P. (2011). In vivo tissue engineering of functional skeletal muscle by freshly isolated satellite cells embedded in a photopolymerizable hydrogel. The FASEB Journal.

[bb0410] Rouwkema J., Khademhosseini A. (2016). Vascularization and angiogenesis in tissue engineering: Beyond creating static networks. Trends in Biotechnology.

[bb0415] Rouwkema J., de Boer J., Van Blitterswijk C.A. (2006). Endothelial cells assemble into a 3-dimensional prevascular network in a bone tissue engineering construct. Tissue Engineering.

[bb0420] Sakaguchi K., Shimizu T., Okano T. (2015). Construction of three-dimensional vascularized cardiac tissue with cell sheet engineering. Journal of Controlled Release.

[bb0425] Sanganalmath S.K., Bolli R. (2013). Cell therapy for heart failure: A comprehensive overview of experimental and clinical studies, current challenges, and future directions. Circulation Research.

[bb0430] Sarugaser R., Lickorish D., Baksh D., Hosseini M.M., Davies J.E. (2005). Human umbilical cord perivascular (HUCPV) cells: A source of mesenchymal progenitors. Stem Cells.

[bb0435] Sathy B.N., Mony U., Menon D., Baskaran V.K., Mikos A.G., Nair S. (2015). Bone tissue engineering with multilayered scaffolds-part I: An approach for vascularizing engineered constructs in vivo. Tissue Engineering. Part A.

[bb0440] Seliktar D., Black R.A., Vito R.P., Nerem R.M. (2000). Dynamic mechanical conditioning of collagen-gel blood vessel constructs induces remodeling in vitro. Annals of Biomedical Engineering.

[bb0445] Shi S., Gronthos S. (2003). Perivascular niche of postnatal mesenchymal stem cells in human bone marrow and dental pulp. Journal of Bone and Mineral Research.

[bb0450] Sims D.E. (2000). Diversity within pericytes. Clinical and Experimental Pharmacology & Physiology.

[bb0455] Stegemann J.P., Nerem R.M. (2003). Phenotype modulation in vascular tissue engineering using biochemical and mechanical stimulation. Annals of Biomedical Engineering.

[bb0460] Swaminathan G., Sivaraman B., Moore L., Zborowski M., Ramamurthi A. (2016). Magnetically-responsive bone marrow mesenchymal stem cell-derived smooth muscle cells maintain their benefits to augmenting elastic matrix neoassembly. Tissue Engineering. Part C, Methods.

[bb0465] Tourovskaia A., Fauver M., Kramer G., Simonson S., Neumann T. (2014). Tissue-engineered microenvironment systems for modeling human vasculature. Experimental Biology and Medicine (Maywood, N.J.).

[bb0470] Traktuev D.O., Merfeld-Clauss S., Li J., Kolonin M., Arap W., Pasqualini R., March K.L. (2008). A population of multipotent CD34-positive adipose stromal cells share pericyte and mesenchymal surface markers, reside in a periendothelial location, and stabilize endothelial networks. Circulation Research.

[bb0475] Tremblay P.L., Hudon V., Berthod F., Germain L., Auger F.A. (2005). Inosculation of tissue-engineered capillaries with the host's vasculature in a reconstructed skin transplanted on mice. American Journal of Transplantation.

[bb0480] Tsai T.N., Kirton J.P., Campagnolo P., Zhang L., Xiao Q., Zhang Z., Xu Q. (2012). Contribution of stem cells to neointimal formation of decellularized vessel grafts in a novel mouse model. The American Journal of Pathology.

[bb0485] Tsang W.P., Shu Y., Kwok P.L., Zhang F., Lee K.K., Tang M.K., Wan C. (2013). CD146 + human umbilical cord perivascular cells maintain stemness under hypoxia and as a cell source for skeletal regeneration. PLoS One.

[bb0490] Valente S., Alviano F., Ciavarella C., Buzzi M., Ricci F., Tazzari P.L., Pasquinelli G. (2014). Human cadaver multipotent stromal/stem cells isolated from arteries stored in liquid nitrogen for 5 years. Stem Cell Research & Therapy.

[bb0495] Vashist A., Ahmad S. (2015). Hydrogels in tissue engineering: Scope and applications. Current Pharmaceutical Biotechnology.

[bb0500] Wanjare M., Kusuma S., Gerecht S. (2013). Perivascular cells in blood vessel regeneration. Biotechnology Journal.

[bb0505] Wanjare M., Kusuma S., Gerecht S. (2014). Defining differences among perivascular cells derived from human pluripotent stem cells. Stem Cell Reports.

[bb0510] Weinberg C.B., Bell E. (1986). A blood vessel model constructed from collagen and cultured vascular cells. Science.

[bb0515] Wendel J.S., Ye L., Tao R., Zhang J., Zhang J., Kamp T.J., Tranquillo R.T. (2015). Functional effects of a tissue-engineered cardiac patch from human induced pluripotent stem cell-derived cardiomyocytes in a rat infarct model. Stem Cells Translational Medicine.

[bb0520] Winkler E.A., Bell R.D., Zlokovic B.V. (2011). Central nervous system pericytes in health and disease. Nature Neuroscience.

[bb0525] Wu Y., Liu G., Chen W., Yang M., Zhu C. (2016). 5-Aminoimidazole-4-carboxamide 1-beta-d-ribofuranoside reduces intimal hyperplasia of tissue engineering blood vessel by inhibiting phenotype switch of vascular smooth muscle cell. Journal of Biomedical Materials Research. Part B, Applied Biomaterials.

[bb0530] Yahagi K., Kolodgie F.D., Otsuka F., Finn A.V., Davis H.R., Joner M., Virmani R. (2016). Pathophysiology of native coronary, vein graft, and in-stent atherosclerosis. Nature Reviews. Cardiology.

[bb0535] Yokoi Y., Namihisa T., Kuroda H., Komatsu I., Miyazaki A., Watanabe S., Usui K. (1984). Immunocytochemical detection of desmin in fat-storing cells (Ito cells). Hepatology.

[bb0540] Zannettino A.C., Paton S., Arthur A., Khor F., Itescu S., Gimble J.M., Gronthos S. (2008). Multipotential human adipose-derived stromal stem cells exhibit a perivascular phenotype in vitro and in vivo. Journal of Cellular Physiology.

[bb0545] Zebardast N., Lickorish D., Davies J.E. (2010). Human umbilical cord perivascular cells (HUCPVC): A mesenchymal cell source for dermal wound healing. Organogenesis.

[bb0550] Zhou M., Qiao W., Liu Z., Shang T., Qiao T., Mao C., Liu C. (2014). Development and in vivo evaluation of small-diameter vascular grafts engineered by outgrowth endothelial cells and electrospun chitosan/poly(epsilon-caprolactone) nanofibrous scaffolds. Tissue Engineering. Part A.

[bb0555] Zieber L., Or S., Ruvinov E., Cohen S. (2014). Microfabrication of channel arrays promotes vessel-like network formation in cardiac cell construct and vascularization in vivo. Biofabrication.

